# Longitudinal Analysis of Physiological Adaptation in Primiparous Dairy Cows During the Transition Period

**DOI:** 10.3390/vetsci13080829

**Published:** 2026-08-19

**Authors:** Akvilė Girdauskaitė, Samanta Grigė, Inga Sabeckienė, Karina Džermeikaitė, Justina Krištolaitytė, Sigitas Japertas, Rokas Macijauskas, Lina Anskienė, Bernadeta Škėlaitė, Ramūnas Antanaitis

**Affiliations:** 1 Large Animal Clinic, Veterinary Academy, Lithuanian University of Health Sciences, Tilžės Str. 18, LT-47181 Kaunas, Lithuaniakarina.dzermeikaite@lsmu.lt (K.D.);; 2 Practical Training and Research Center, Lithuanian University of Health Sciences, Topolių g. 6, LT-54310 Kaunas, Lithuania; 3 Department of Animal Breeding, Faculty of Animal Sciences, Lithuanian University of Health Sciences, Tilžės 18, LT-47181 Kaunas, Lithuania

**Keywords:** metabolic adaptation, transition period, primiparous dairy cows, blood biomarkers, rumen sensors, precision monitoring

## Abstract

The transition period is a physiologically demanding stage in primiparous dairy cows, as the onset of lactation occurs simultaneously with continued growth. This longitudinal study initially enrolled 22 primiparous Holstein dairy cows. Eight cows that developed transition disorders during the monitoring period were excluded according to the predefined study protocol. Therefore, the final analysis included 14 clinically healthy primiparous Holstein dairy cows monitored from four weeks before calving to five weeks postpartum to characterize normal physiological adaptation during the transition period. Cows were monitored using continuous intraruminal sensors and automated milk analysis systems, while blood samples were collected weekly and analysed using linear mixed models with repeated measurements. Milk yield increased from 17.9 kg/day in week +1 to 26.3 kg/day in week +5 (a relative increase of 47%). Significant temporal changes were observed in iron (Fe), phosphorus (P), glucose (GLUC), triglycerides (TRIG), urea (UREA), albumin (ALB), and alanine aminotransferase (ALT). Serum Fe decreased from 30.9 µmol/L at week −2 to 14.5 µmol/L at week +2 (−53%), while P declined from 2.95 mmol/L at week +1 to 1.41 mmol/L at week +2 (−52%). Sensor data showed reduced physical activity (−59%) and increased water intake from 60.6 L/day in week −4 to 99.4 L/day in week +1, whereas rumination time and rumen pH remained relatively stable. Correlation analysis identified significant associations among milk yield, metabolic variables, and behavioural indicators. These findings suggest coordinated physiological adaptation during the transition period and support the use of combined blood and sensor-based monitoring to better understand early-lactation dynamics in primiparous dairy cows.

## Simple Summary

This study investigated week-by-week physiological changes during the transition period (4 weeks before to 5 weeks after calving) in 14 clinically healthy primiparous Holstein dairy cows. Primiparous dairy cows must sustain both milk production and continued growth, making this stage particularly demanding. Following calving, milk production increased by 47%, physical activity decreased by nearly 60%, and water intake increased by 64%, from 60.6 L/day before calving to 99.4 L/day during early lactation. At the same time, several blood indicators changed markedly: iron and serum phosphorus concentrations decreased by more than 50%, and glucose levels declined during early lactation, reflecting increased metabolic demand. By combining blood biochemical data with continuous sensor-based monitoring, this study provides additional insight into physiological adaptation during early lactation. These findings highlight the potential value of integrating traditional laboratory measurements with precision monitoring technologies to improve the monitoring and management of the transition period in dairy herds.

## 1. Introduction

Dairy cows experience major physiological and metabolic changes during the transition period. Energy and nutrient requirements increase rapidly to support fetal growth, mammary gland development, colostrum formation, and the onset of lactation, while feed intake often decreases during the last week before calving. Dry matter intake (DMI) typically declines by approximately 10–30% before calving, whereas energy demand increases sharply, contributing to the development of negative energy balance in early-lactation dairy cows [1]. During the final weeks of gestation, the mammary gland undergoes rapid structural and functional development, including increased blood flow, growth of secretory tissue, and visible udder enlargement in preparation for lactation [2]. It is now recognised that these adaptations are not limited to the traditional three weeks before and after calving, but may begin earlier in late gestation and continue for several weeks after calving [2,3].

Blood biochemical parameters, such as circulating non-esterified fatty acids (NEFA) and other energy-related metabolites, provide insight into the degree of energy mobilisation and metabolic status as cows progress through late gestation and early lactation [4]. NEFA concentrations typically increase from approximately 0.2–0.3 mmol/L prepartum to 0.5–0.7 mmol/L postpartum, reflecting lipid mobilization during negative energy balance, while blood glucose concentrations tend to decrease during early lactation [2,5]. Numerous studies highlight that monitoring blood biomarkers such as NEFA, glucose, urea, TRIG, and mineral parameters at repeated time points throughout the transition period captures temporal dynamics that reflect ongoing physiological change [6,7]. Similarly, variation in protein metabolism biomarkers, particularly urea, total protein, and albumin, over the transition period has been shown to relate to energy partitioning and nutrient utilisation, emphasising the value of serial biochemical monitoring [8].

Milk composition traits—including fat, protein, and lactose concentrations—also evolve markedly during early lactation and are closely tied to the metabolic state of the cow [9]. The milk fat-to-protein ratio (FPR) in particular has been investigated as a non-invasive indicator of energy balance, with higher ratios corresponding to greater mobilisation of body reserves in cows experiencing negative energy balance [10,11]. A FPR of approximately 1.2–1.4 is generally considered physiologically normal, whereas values above 1.4 are associated with negative energy balance and lipid mobilization, and values below 1.0 may indicate rumen acidosis [8,9]. Meta-analytical evidence further suggests that milk composition indicators such as FPR and milk yield are associated with digestive function and rumen fermentation patterns, providing additional physiological context when interpreted alongside behavioural and biochemical measures [3].

In recent years, intraruminal boluses have become an important tool for continuously monitoring dairy cows during the transition period. Once administered, these gravity-retained devices remain in the reticulorumen and record parameters such as temperature and pH over extended periods without the need for repeated handling of the animals [10]. Continuous reticulorumen temperature measurements have been shown to reflect changes in drinking behavior and feed intake around calving, providing insight into physiological adjustments occurring during this demanding phase [5,11]. Normal reticulorumen temperature in dairy cows typically ranges from approximately 38.5 to 40.0 °C. Temperatures above 40.5 °C may indicate fever or inflammatory processes, whereas temperatures below 38.0 °C may be associated with reduced feed intake or drinking events [11,12]. Similarly, studies evaluating rumen temperature and related sensor data demonstrate their usefulness in identifying shifts in digestive activity and metabolic adaptation [11]. Modern bolus systems may also incorporate motion-sensitive components that enable indirect assessment of rumination and activity patterns, allowing researchers to follow week-by-week changes in behavior and rumen function during late gestation and early lactation. Together, these technologies provide a practical and objective way to describe physiological adaptation across the transition period under routine farm conditions [12,13].

Studies assessing blood metabolites, milk composition, and sensor-derived parameters during the transition period are available, but these indicators are often evaluated separately [14]. Integrating longitudinal blood biochemical data, milk composition, and continuous intraruminal sensor measurements from the same animals may provide a more comprehensive understanding of physiological adaptation during early lactation. Primiparous cows represent a particularly important group due to their continued growth and different metabolic demands compared with multiparous cows [15,16].

Because transition disorders profoundly influence metabolic, biochemical, and behavioural variables, characterization of physiological adaptation requires evaluation of clinically healthy animals. Several previous studies investigating metabolic adaptation have therefore deliberately restricted analyses to healthy dairy cows to establish physiological reference patterns rather than disease-associated responses [1,17]. Therefore, the aim of the present study was to characterise week-by-week changes in blood biochemical parameters, milk production and composition, and sensor-derived physiological and behavioural indicators from four weeks before calving to five weeks postpartum in primiparous dairy cows. This integrative approach enables the identification of coordinated responses across multiple physiological systems during the transition period. Furthermore, by focusing specifically on primiparous cows and analysing week-by-week dynamics, this study provides detailed insight into early-lactation adaptation in a group with distinct metabolic demands that remains relatively underrepresented in the literature.

## 2. Materials and Methods

### 2.1. Housing Conditions of the Study Animals

All procedures involving animals were carried out in accordance with the Lithuanian Law on Animal Welfare and Protection and were approved under permit No. G2-298. The study was conducted on a commercial dairy farm in Lithuania with approximately 119 lactating Holstein cows and lasted from April 2025 to January 2026. A total of 22 primiparous Holstein cows were initially enrolled in the study, and 14 clinically healthy cows were included in the final longitudinal analysis. The objective of the study was to characterize normal physiological adaptation during the transition period. Therefore, the analytical cohort was restricted to clinically healthy cows that remained free of transition disorders throughout the entire observation period. This approach was chosen to minimize the confounding effects of disease-related metabolic alterations and to allow characterization of physiological rather than pathological adaptation, consistent with previous longitudinal studies investigating metabolic adaptation in clinically healthy dairy cows [17,18]. The average body weight of the cows included in the study was 500 ± 45 kg. The average age of the cows at calving was 24.2 ± 1.7 months, and the mean body condition score before calving was 3.25 ± 0.25 (5-point scale). Clinically healthy cows were defined as animals showing no signs of systemic or reproductive disease during the study period and requiring no veterinary treatment. Health status was monitored daily through routine clinical examinations, including rectal temperature assessment and general clinical evaluation. In addition, somatic cell count and milk electrical conductivity were continuously monitored using the automatic robotic milking system, while rumination time, reticulorumen pH, reticulorumen temperature, and the milk FPR were continuously monitored using the automated herd management systems. These indicators were evaluated together to identify cows developing transition disorders. All cows belonged to the same Holstein herd and were managed under identical housing, feeding, and herd management conditions before and throughout the study period. As all animals were primiparous, no previous lactation-related variability was present.

The cows were housed in a mechanically ventilated free-stall barn with free access to drinking water and were milked using an automatic robotic milking system (DeLaval International AB, Tumba, Sweden). According to farm records, the average annual milk yield was 9991 kg per cow, with an average fat content of 3.94% and protein content of 3.41%.

During the study period, cows were fed total mixed rations (TMR) formulated according to the farm’s standard nutritional management protocol to meet the nutritional requirements of dairy cows before and after calving. The TMR consisted of approximately 31% maize silage, 10% grass silage, 4% alfalfa hay, 49% grain concentrate mash, and 6% mineral supplement. The chemical composition of the ration included 50.7% dry matter, 15.8% crude protein, 28.3% neutral detergent fibre, 19.8% acid detergent fibre, 38.7% non-fibre carbohydrates, and a net energy for lactation of 1.6 Mcal/kg. Feed was offered twice daily and was available ad libitum throughout the study period. Feed refusals were removed daily before the morning feeding to maintain feed freshness and ensure unrestricted access.

### 2.2. Experimental Grouping

Initially, 22 primiparous Holstein cows were prospectively enrolled and monitored throughout the transition period. According to the predefined study protocol, only cows that remained clinically healthy throughout the entire observation period and had complete longitudinal data were included in the final analysis. During the monitoring period, eight cows did not meet these inclusion criteria due to the development of transition disorders, including subclinical mastitis (n = 4), ketosis (n = 2), lameness (n = 1), and metritis (n = 1). Following the diagnosis of a transition disorder, the affected cows were transferred to a separate management group according to the farm’s routine management protocol. Consequently, automated sensor and milk recording data were no longer available, and no further blood samples were collected after disease diagnosis. Therefore, these animals were excluded from the final longitudinal analysis. The final analytical cohort consisted of 14 cows. This selection strategy was intended to ensure that the observed longitudinal changes reflected normal physiological adaptation rather than disease-associated metabolic alterations.

Each animal was monitored for nine consecutive weeks, encompassing four weeks before calving and five weeks after parturition. This duration was selected to capture the transition period and the initial phases of lactation. Health status was assessed weekly throughout the observation period. Animals that developed clinical or subclinical disorders known to influence metabolic adaptation, including mastitis, ketosis, metritis, lameness, displaced abomasum, digestive disorders, or other systemic diseases, did not meet the predefined inclusion criteria for the final analysis. Clinical examinations were performed weekly by the same veterinarian using standardized clinical assessment procedures. Ketosis was diagnosed based on clinical signs together with elevated blood β-hydroxybutyrate concentrations (>1.2 mmol/L). Subclinical mastitis was identified using somatic cell count data (>200,000 cells/mL) in the absence of visible clinical signs, whereas clinical mastitis was diagnosed based on abnormal milk appearance and clinical evidence of udder inflammation. Metritis was diagnosed according to the presence of abnormal uterine discharge combined with systemic clinical signs after calving. Lameness was assessed during routine locomotion scoring and clinical examination.

Measurements were performed once weekly at a consistent time of day to minimize diurnal variation. Clinical examinations were conducted each morning by the same veterinarian to ensure consistency.

### 2.3. Blood Sample Collection and Biochemical Analysis

Blood samples were collected once weekly throughout the entire study period. To reduce potential diurnal variation, sampling was consistently performed at 10:00 a.m. on the same weekday each week, approximately 2 h after the morning feeding. Because cows were milked voluntarily using an automatic robotic milking system, the interval between blood sampling and the most recent milking event was not standardized. Sterile evacuated serum tubes without anticoagulant (BD Vacutainer^®^, Becton Dickinson, Eysins, Switzerland) were used to draw about 10 mL of blood from the coccygeal vein. Following collection, samples were carefully transported to the Laboratory of Clinical Tests at the Lithuanian University of Health Sciences’ Large Animal Clinic of the Veterinary Academy. Blood samples were processed shortly after collection and centrifuged within approximately 1–2 h at 1500× *g* for 15 min to separate serum.

Serum biochemical parameters were measured using an automated wet chemistry analyzer (RX Daytona, Randox Laboratories Ltd., Crumlin, UK) with manufacturer-specific reagent kits. Internal quality control procedures and routine calibration were performed in accordance with the manufacturer’s recommendations to ensure analytical precision and reliability. The analysed variables included ALT, AST, gamma-glutamyl transferase (GGT), calcium (Ca), magnesium (Mg), P, Fe, creatinine (CREA), ALB, GLUC, total protein (TP), TRIG, urea, and NEFA.

### 2.4. Continuous Monitoring of Behavioral, Physiological, and Feeding Parameters

In all 14 cows included in the study, continuous monitoring of physiological and behavioral parameters was performed from four weeks before calving until five weeks postpartum. Reticulorumen temperature, pH, rumination time (RT), physical activity, and water intake were recorded using intraruminal SmaXtec boluses (SmaXtec animal care technology^®^, Graz, Austria). Physical activity was expressed as activity units generated by the SmaXtec system. These units represent a proprietary dimensionless index derived from the bolus motion sensor and reflect the relative intensity of cow movement over time, with higher values indicating greater activity. Approximately four weeks before the expected calving date, the heifers were moved to the close-up group equipped with the automatic robotic milking system as part of the farm’s standard transition management protocol. During this prepartum period, the animals voluntarily visited the robotic unit to receive concentrate feed and adapt to the lactation ration, although they were not milked before calving. Individual daily concentrate intake (kg/day) was automatically recorded by the feeding module integrated into the automatic robotic milking system (DeLaval International AB, Tumba, Sweden) throughout both the prepartum and postpartum periods. The milk FPR was calculated as milk fat percentage (%) divided by milk protein percentage (%) for each milk recording obtained from the automatic milking system. Behavioural, physiological, and feeding-related parameters were monitored longitudinally throughout the transition period using an approach similar to that previously described in dairy cows for the evaluation of metabolic adaptation around calving [19,20].

Each cow received a single bolus approximately five weeks before the expected calving date to allow adequate sensor stabilization prior to the start of the observation period. Before administration, the bolus was activated, assigned to the respective cow by linking it to the ear tag identification number, and connected to the central monitoring system.

Boluses were administered orally using a dedicated applicator according to the manufacturer’s instructions. During placement, cows were restrained in self-locking head gates, and the head was gently stabilized to ensure correct positioning at the base of the tongue. The devices are designed to remain permanently in the reticulum. In order to detect any possible negative effects, animals were observed for two hours after bolus administration.

Receiving antennae were used to wirelessly transfer data to the SmaXtec system, where an internal computer processed the signals, an analog-to-digital converter translated them, and an inbuilt memory chip stored them. Measurements were recorded at 10 min intervals throughout the study. Data acquisition, processing, and visualization were performed using SmaXtec Messenger^®^ software (version 4; SmaXtec animal care GmbH, Graz, Austria; https://www.smaxtec.com/, accessed on 12 May 2026), enabling continuous tracking of all monitored parameters for each cow during the entire experimental period.

### 2.5. Automated Recording of Milk Parameters

Milk performance was followed in all cows from the first day after parturition. Milk yield referred to total daily milk production (kg/day). Cows were milked voluntarily using an automatic robotic milking system with multiple milking visits per day. Individual daily milk yield, together with milk fat, protein, lactose content, and the FPR, was automatically recorded using an in-line milk analysis system (Brolis Sensor Technology, Vilnius, Lithuania).

To ensure comparability between metabolic and production data, the milk composition values included in the statistical analysis were obtained from the same day on which blood samples were collected and did not represent weekly averages. The analyzer is based on near-infrared spectroscopy (2100–2400 nm) employing a tunable GaSb external cavity laser. As milk passed through the system during milking, its flow was continuously measured, and spectral data were collected to determine the concentration of the main milk components.

The sensor was permanently installed within the robotic milking unit of the automatic robotic milking system, allowing uninterrupted measurement without additional reagents or servicing procedures. Milk constituents were assessed every five seconds, and session-level values were calculated as flow-adjusted averages, providing representative composition data for the entire milking event of each cow.

### 2.6. Statistical Analysis

All statistical analyses were performed using IBM SPSS Statistics (Version 31, IBM Corp., Armonk, NY, USA; https://www.ibm.com/spss, accessed on 12 May 2026). The final dataset consisted of repeated measurements collected from the same cohort of 14 clinically healthy primiparous dairy cows from Week −4 before calving to Week +5 after calving. All statistical analyses were performed using this final analytical cohort. Continuous variables included milk yield, milk composition traits (fat, protein, lactose, FPR), blood biochemical parameters (ALT, AST, GGT, Ca, Mg, P, Fe, CREA, ALB, GLUC, TP, TRIG, UREA, NEFA), sensor-based indicators (rumen temperature, rumination time, activity, water intake, rumen pH), and concentrate intake.

Prior to inferential analyses, descriptive statistics (mean, standard deviation, minimum, maximum, and 95% confidence intervals) were calculated, and distribution diagnostics were performed using the Shapiro–Wilk test for each variable within each week. Several biochemical indicators showed right-skewed distributions; therefore, ALT, NEFA, TRIG, UREA, and P were log10-transformed prior to analysis to improve normality and stabilize variances. Variables approximating normal distribution were analysed on their original scale. For clarity and biological interpretation, results for log-transformed variables are presented as back-transformed values in the original measurement units.

Data were organised in a long format, where each row represented one cow at a specific week of measurement. No missing observations occurred during the study, and complete longitudinal data were available for all 14 cows included in the final analytical cohort. Repeated measurements were analysed using linear mixed models (LMM), with WEEK (−4, −3, −2, −1, +1, +2, +3, +4, +5) included as a fixed effect and Cow ID as a random intercept to account for repeated observations within animals. An autoregressive covariance structure [AR(1)] was selected a priori because measurements obtained closer in time within the same cow were expected to be more strongly correlated than measurements obtained further apart. Alternative covariance structures were not formally compared using information criteria such as AIC or BIC. Therefore, all repeated measurements were included in the linear mixed models without the need for data imputation or special procedures for handling missing data.

The longitudinal data were analysed using a linear mixed-effects model of the following form: Y_ij = μ + Week_i + Cow_j + ε_ij, where Y_ij represents the dependent variable measured in cow j at week i, μ is the overall mean, Week_i—week is included as a fixed effect (categorical factor with 9 levels), Cow_j—cow is included as a random intercept to account for repeated measurements within animals, and ε_ij represents the residual error term.

Estimated marginal means were calculated for each week, and pairwise comparisons were adjusted using the Sidak correction. Statistical significance was set at *p* < 0.05, while *p* < 0.01 and *p* < 0.001 were considered highly significant.

Milk production traits (milk yield, fat, protein, lactose, and FPR) were analysed for postpartum weeks only (+1 to +5), whereas blood biochemical, rumen, and behavioural variables were analysed across all weeks from −4 to +5 relative to calving. All milk production variables were analysed using weekly mean values derived from the same sampling days used for blood biochemical analyses.

Within-cow associations among variables were evaluated using repeated-measures correlation analysis. To ensure methodological consistency, variables that were log10-transformed for the primary analyses (ALT, NEFA, TRIG, UREA, and P) were also used in their transformed form in the correlation analysis. Repeated-measures correlations were calculated using within-cow centred (_wc) values, thereby accounting for both non-normal distributions and the longitudinal structure of the data. This approach ensured that correlation coefficients reflected within-cow associations independent of between-animal variability.

Data were screened using graphical methods (boxplots and residual plots) for outliers and data entry errors prior to analysis. No extreme outliers requiring exclusion were identified. Model assumptions were evaluated using residual diagnostics, including assessment of normality (Q–Q plots) and homogeneity of variance. Model fit was assessed based on residual behaviour and the adequacy of the selected covariance structure.

## 3. Results

### 3.1. Changes in Milk Yield and Composition Across Postpartum Weeks (+1 to +5)

Milk yield increased significantly across postpartum weeks (Table 1). Milk yield in week +5 was 47.2% higher than in week +1 (*p* < 0.01), whereas no significant differences were observed among the remaining postpartum weeks.

Milk composition also changed during early lactation (Table 1). Both milk fat and protein percentages declined progressively after calving, with the highest values observed in week +1 and the lowest in week +5. In contrast, lactose percentage remained stable throughout the study period (*p* > 0.05).

### 3.2. Changes in Blood Biochemical Parameters from Prepartum Week −4 to Postpartum Week +5

ALT activity showed only minor variation across weeks, whereas calcium concentrations differed only between weeks −2 and +3 (Table 2).

Serum iron exhibited the most pronounced temporal changes, with the highest concentrations observed before calving and significantly lower values throughout the postpartum period (Table 2).

Glucose concentrations gradually declined after calving and reached their lowest values in week +4. Triglyceride concentrations also decreased around calving, whereas urea increased markedly between weeks +1 and +2.

Serum phosphorus showed a pronounced decline between weeks +1 and +2 before gradually recovering. In contrast, AST, Mg, NEFA, and TP remained relatively stable throughout the observation period, with no significant differences between weeks (*p* > 0.05).

### 3.3. Changes in Rumen Physiology, Rumination, Activity, and Water Intake from Prepartum Week −4 to Postpartum Week +5

Rumen temperature showed only minor variation across the study period (Table 3), with a significant difference detected only between weeks −1 and +4. Although the linear mixed model indicated significant overall effects of week on rumination time and rumen pH, no statistically significant pairwise differences between individual weeks were identified after Sidak adjustment.

In contrast, physical activity declined markedly after calving, with significantly higher prepartum values than during the postpartum period. Water intake showed the opposite pattern, increasing substantially after calving and remaining elevated throughout early lactation.

Concentrate intake increased progressively across the transition period, with significantly higher values observed during the later postpartum weeks than before calving.

### 3.4. Linear Mixed Model Summary for All Dependent Variables

The linear mixed model demonstrated a significant effect of week on several milk production traits (Table 4). Milk yield, milk fat percentage, and milk protein percentage varied significantly across weeks, whereas lactose percentage and the FPR were not significantly affected.

Among blood biochemical parameters, significant week effects were observed for ALB, ALT, iron, GLUC, Mg, P, TP, TRIG, and UREA. In contrast, AST and NEFA did not vary significantly across weeks, whereas Ca and CREA showed borderline effects.

Among rumen and behavioural variables, week significantly affected rumen temperature, rumination time, rumen pH, concentrate intake, activity, and water intake.

AR(1) correlation estimates varied among variables, ranging from −0.26 to 0.74. Positive estimates indicate stronger similarity between measurements obtained closer in time, whereas estimates close to zero indicate weak temporal dependence. Negative estimates indicate weak inverse temporal correlation and should be interpreted with caution, particularly given the limited sample size.

### 3.5. Selected Repeated-Measures Correlations Among Milk Production, Metabolic, and Behavioural Variables

**Figure 1:**
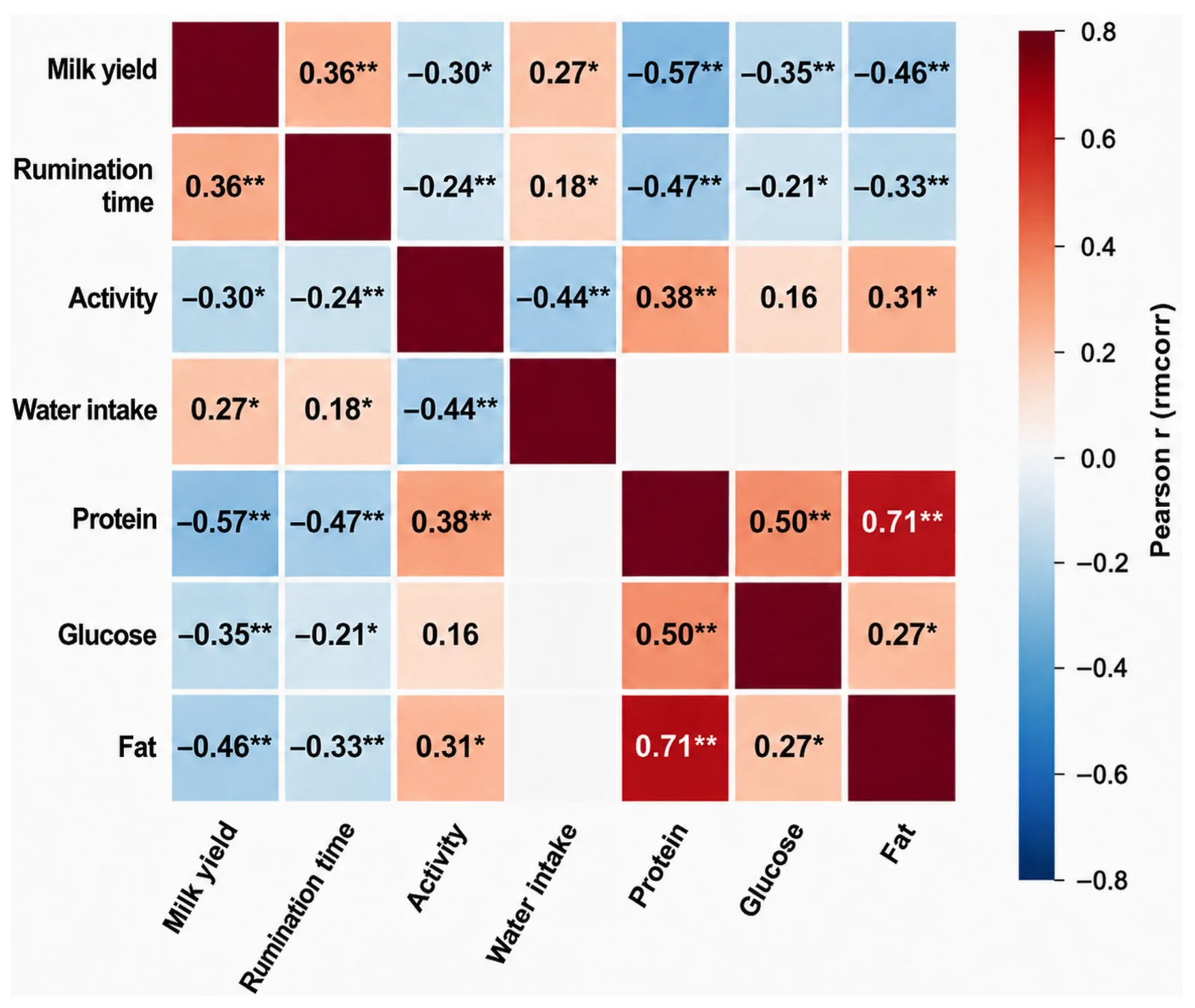
Heat map of the strongest statistically significant repeated-measures (within-cow) correlations among selected milk production, behavioural, and metabolic variables during the transition period. Only correlations with |r| ≥ 0.30 and *p* < 0.05 are shown. Cell values represent repeated-measures correlation coefficients (rmcorr, r), with statistical significance indicated (* *p* < 0.05; ** *p* < 0.01). Color intensity reflects the strength and direction of the correlations (blue = negative; red = positive).

Repeated-measures correlation analysis identified several statistically significant within-cow associations among milk production, behavioural, and metabolic variables during the transition period (Figure 1). Milk yield showed a positive within-cow association with rumination time (r = 0.36, *p* < 0.01) and water intake (r = 0.27, *p* < 0.05), while a negative association was observed with physical activity (r = −0.30, *p* < 0.05). In addition, milk yield was negatively associated with milk protein content (r = −0.57, *p* < 0.01), glucose concentration (r = −0.35, *p* < 0.01), and milk fat content (r = −0.46, *p* < 0.01).

Regarding milk composition and metabolic indicators, milk protein content showed strong positive within-cow associations with glucose concentration (r = 0.50, *p* < 0.01) and milk fat content (r = 0.71, *p* < 0.01). Behavioural variables were also interrelated, as physical activity was negatively associated with rumination time (r = −0.24, *p* < 0.01) and water intake (r = −0.44, *p* < 0.01). All reported associations represent within-cow relationships after accounting for repeated measurements within animals and should not be interpreted as evidence of causal relationships.

Additional within-cow correlations involving biochemical and rumen variables, generally with weaker effect sizes, are presented in Appendix A.

## 4. Discussion

This study aimed to evaluate how milk production, metabolic parameters, and behavioural indicators change together in clinically healthy primiparous dairy cows during the transition period. The results showed that increased milk production after calving was accompanied by parallel changes in blood biochemical and behavioural variables, consistent with coordinated physiological adaptation to early lactation. Similar coordinated physiological and metabolic adaptations during the transition period have also been reported previously in dairy cows adapting to the onset of lactation [2]. The most pronounced alterations were observed after calving, reflecting the substantial metabolic reorganization required as cows transition from gestation to established lactation.

Milk yield increased rapidly during early lactation, which is characteristic of the ascending phase of the lactation curve and reflects the prioritization of nutrient partitioning toward milk synthesis immediately after calving [2,12]. At the same time, milk fat and protein percentages declined. This inverse relationship between milk yield and component concentration is widely reported and is partly explained by dilution effects, whereby increasing milk volume reduces component percentages even when total component yield increases [3]. In addition, transient changes in amino acid and energy availability during early lactation may influence protein synthesis efficiency in the mammary gland [20,21,22]. These changes likely reflect the rapid increase in energy demand after calving. At the same time, dry matter intake typically remains limited during early lactation in primiparous cows, resulting in a transient imbalance between nutrient supply and metabolic requirements [2,23].

In contrast, lactose concentration remained comparatively stable, rising only slightly from 4.62% in week +1 to 4.74% in week +5. Because lactose is the primary osmotic regulator of milk volume and depends directly on glucose supply to the mammary gland, its stability suggests that systemic GLUC availability was sufficient to sustain lactose synthesis despite increasing milk output [4,24]. Approximately one-fifth of circulating glucose is directed toward lactose production during lactation, underlining its central role in determining milk volume [25]. The relative constancy of lactose therefore indicates that glucose supply was maintained at a level sufficient to support milk synthesis during early lactation.

Glucose concentrations further reflect this metabolic adjustment. Values were higher before calving and declined postpartum, reaching the lowest level at week +4. Such a decrease is physiologically expected, as glucose demand increases sharply after calving to support lactose synthesis and other anabolic processes [26]. In ruminants, glucose is produced primarily through hepatic gluconeogenesis rather than direct absorption, and early lactation places considerable pressure on this pathway [27]. Moderate reductions in circulating glucose are therefore more likely to reflect increased mammary uptake than systemic deficiency [28].

In contrast to typical early-lactation patterns, NEFA concentrations did not show significant changes in this study. Under normal conditions, NEFA levels increase after calving due to negative energy balance and mobilization of body fat reserves [6,26]. The absence of this pattern may suggest that the degree of negative energy balance was relatively limited in the study population. However, this interpretation should be made with caution because overall energy balance was not directly evaluated. Several methodological factors should also be considered. Individual total DMI was not measured. Individual concentrate intake was recorded throughout the study and increased significantly after calving. However, this primarily reflected the farm’s feeding strategy, in which concentrate allowance was gradually increased during early lactation. Such a strategy is commonly implemented to meet the increasing energy demands associated with milk production while facilitating adaptation to higher-energy diets [29]. Because forage intake, which represents the major proportion of total DMI, was not quantified, total nutrient intake and the roughage-to-concentrate ratio could not be determined. Similarly, changes in body weight and body condition score were not assessed, further limiting the interpretation of the observed NEFA responses. Consequently, the observed increase in concentrate intake should not be interpreted as evidence of voluntary changes in total feed intake or diet selection by the cows. In addition, weekly blood sampling may not have captured short-term fluctuations in NEFA concentrations, which are known to vary rapidly during early lactation [6,14,27]. Because the present study focused on this selected population, the observed NEFA responses likely represent successful physiological adaptation rather than the broader range of metabolic responses expected in the overall transition dairy cow population. Therefore, the observed stability should not be interpreted as indicating the absence of metabolic adaptation but rather as reflecting successful physiological adaptation in the study population. Similar variability in NEFA responses during the transition period has also been described previously, particularly in cows maintained under good management conditions and without clinical disease, indicating that metabolic adaptation during early lactation may differ substantially between herds and individuals [30].

Urea concentrations showed a distinct temporal pattern, with a sharp increase between weeks +1 and +2 followed by a gradual decline. Circulating urea reflects hepatic nitrogen metabolism and the balance between ruminal ammonia production and microbial protein synthesis. During early lactation, dry matter intake increases rapidly, and shifts in rumen fermentation may temporarily affect nitrogen utilization efficiency [31]. The marked increase observed in this study may reflect changes in nitrogen metabolism associated with the transition to lactation and the increasing dietary protein supply required to support milk production. Blood urea concentrations are influenced by the balance between dietary protein and energy supply, as well as ruminal nitrogen utilization and hepatic urea synthesis [32,33]. Therefore, transient increases in urea concentration during early lactation may represent physiological adaptation rather than impaired metabolic function.

Mineral metabolism also changed noticeably during the transition period. Serum phosphorus concentrations declined markedly around week +2 postpartum before gradually stabilizing. P is essential for cellular energy metabolism and ATP production, and its demand increases rapidly after calving because large amounts are required for milk synthesis. One possible explanation is the increased physiological demand for P during early lactation, including P secretion into milk [34]. Similar decreases have been reported previously in early-lactation dairy cows, reflecting the high metabolic demands that occur immediately after calving [35,36]. However, because individual dry matter intake, dietary phosphorus intake, and milk phosphorus output were not measured, the mechanism underlying the observed decrease could not be confirmed in the present study. Although phosphorus concentrations decreased substantially, values generally remained within or close to physiological ranges reported for transition dairy cows [31,35]. None of the cows showed clinical signs typically associated with severe P deficiency, such as recumbency or severe depression. However, insufficient P availability during early lactation may negatively affect feed intake, energy metabolism, and milk production, particularly if reductions are prolonged [31,36]. These findings support the importance of adequate mineral balance during the transition period.

Serum iron concentrations also declined considerably after calving. Iron is important for oxygen transport and cellular metabolism. Serum iron concentrations may change during early lactation as part of physiological and inflammatory adaptations occurring around parturition [2,37,38]. Similar postpartum decreases have been described previously in clinically healthy dairy cows [39]. Despite the marked reduction observed in the present study, no clinical signs suggestive of anemia were detected in the animals. Previous studies have suggested that more severe or prolonged reductions in iron concentrations may impair immune function, oxygen transport, and productive performance [37,39]. However, inflammatory markers, hematological variables, and hepcidin concentrations were not evaluated in the present study; therefore, the physiological mechanisms underlying the observed iron changes cannot be confirmed directly.

Behavioural indicators derived from continuous sensor monitoring provided additional evidence of coordinated adaptation. Physical activity decreased markedly after calving and remained consistently lower throughout the postpartum period. This decline occurred alongside increasing milk production and may reflect behavioural adaptation during early lactation. Reduced locomotor activity during early lactation has been widely reported and is often interpreted as a strategy to conserve energy for milk synthesis and metabolic adaptation [40,41,42]. In primiparous cows, this response may be more pronounced, as nutrients must support both milk production and ongoing growth [9,43]. The observed reduction in activity may reflect physiological adaptation to early lactation. However, because direct welfare indicators were not evaluated, interpretations regarding animal welfare should be made cautiously.

Conversely, water intake increased substantially after calving, in parallel with rising milk yield. Water consumption in dairy cows is closely associated with milk production, dry matter intake, and physiological demand, and typically increases during early lactation [44]. Because milk contains approximately 87% water, even moderate increases in production require additional water intake. Environmental temperature was not recorded in this study and may have contributed to variation in water intake, particularly during warmer periods. Nevertheless, the observed association between milk yield and water intake is consistent with physiological adaptation during early lactation. However, this association should not be interpreted as evidence of a causal relationship. Environmental and management conditions may additionally influence drinking behaviour during early lactation, particularly under conditions of heat stress or altered feeding patterns [45].

Rumen temperature also showed a statistically significant overall effect of week; however, the observed changes were small and values remained within the physiological range throughout the study period. The slightly higher temperature before calving compared with week +4 may reflect normal variation associated with changes in feed intake, drinking behaviour, and rumen fermentation during the transition period [11,12].

Although the linear mixed model indicated a significant overall effect of week, no significant pairwise differences in rumination time were detected after multiple-comparison adjustment. Rumination is closely linked to fibre intake, rumen function, and overall health, and reductions are commonly observed in cows experiencing metabolic or inflammatory stress [46]. In the present study, rumination time showed a gradual numerical increase throughout the observation period, which may reflect progressive adaptation to increasing feed intake during early lactation. However, because individual forage intake was not measured, this interpretation remains speculative. Similarly, although rumen pH was significantly affected by week in the linear mixed model, no significant pairwise differences were detected, suggesting that rumen acid–base conditions were generally maintained throughout the transition period.

When considered together, the observed changes in milk production, metabolic indicators, and behavioural parameters reveal a coordinated pattern of physiological adaptation. The increase in milk yield was accompanied by shifts in glucose and mineral metabolism, reduced physical activity, and increased water intake, consistent with coordinated physiological adaptation during early lactation. In primiparous cows, this coordination is particularly important, as nutrients must be allocated simultaneously to milk production, maintenance, and ongoing growth [6,47,48]. Evaluating multiple parameters simultaneously therefore provides a more comprehensive understanding of transition dynamics than assessing individual systems in isolation.

An important feature of the present study is that the analysis was restricted to clinically healthy primiparous cows. This approach was chosen to evaluate physiological adaptation during the transition period without the confounding influence of transition disorders, which are known to substantially affect metabolic, biochemical, and behavioural responses. Similar study designs have been used to establish physiological reference patterns during early lactation in healthy dairy cows [17,18]. Therefore, the temporal changes observed in the present study should be interpreted as representing normal physiological adaptation rather than the full biological variability of the overall transition dairy cow population. Furthermore, potential confounding factors related to parity, housing conditions, feeding regimen, and management practices were minimized because all animals originated from a single herd managed under uniform conditions.

This study has several limitations. First, the relatively small sample size may have reduced statistical power, particularly for variables showing high inter-animal variability. Accordingly, statistically significant findings should be interpreted together with their biological relevance, particularly for variables showing relatively small temporal changes. However, the sample size is comparable to that used in previous longitudinal transition-period studies involving intensive repeated physiological and metabolic monitoring. For example, Ghaffari et al. [18] investigated longitudinal metabolic adaptations in 12 Holstein dairy cows using repeated sampling throughout the transition period. Furthermore, individual total dry matter intake (DMI) was not measured. Although individual concentrate intake was recorded, forage intake, which constitutes the major proportion of total DMI, was not quantified. Consequently, total nutrient intake and energy balance could not be fully assessed. In addition, only clinically healthy cows that remained free of transition disorders throughout the observation period were included in the final analysis. This study design was intended to characterize normal adaptation during the transition period; however, it limits the external validity of the findings. Therefore, the present findings describe physiological adaptation under the conditions of this study and should not be generalized to the entire transition dairy cow population. Blood samples were collected weekly and therefore may not have captured short-term metabolic fluctuations. Environmental conditions (e.g., temperature, humidity, and temperature–humidity index) were not recorded. Therefore, their potential influence on water intake, physical activity, feed intake, and metabolic responses could not be evaluated. Finally, because the study was conducted on a single commercial dairy farm, caution is warranted when extrapolating these findings to other management systems. Nevertheless, the repeated-measures longitudinal design enabled detailed characterization of coordinated physiological, metabolic, and behavioural adaptations throughout the transition period.

## 5. Conclusions

This study characterized temporal changes in milk production, blood biochemical parameters, and behavioural indicators during the transition period in clinically healthy primiparous dairy cows. The main findings included increased milk yield after calving, changes in glucose and mineral metabolism, reduced physical activity, and increased water intake, whereas rumination time and rumen pH remained relatively stable. Together, these observations provide additional insight into the physiological changes occurring during early lactation in clinically healthy primiparous cows.

Evaluating physiological parameters in combination, rather than individually, may provide a more comprehensive understanding of transition dynamics. Integrating continuous sensor-derived information with longitudinal blood biochemical measurements may improve monitoring of physiological changes during early lactation.

Weekly monitoring of these parameters may improve the identification of temporal patterns of physiological adaptation and support herd management during the transition period. However, because this study included a relatively small, selected group of clinically healthy primiparous cows from a single commercial dairy herd, the findings describe physiological patterns observed under these study conditions and should not be generalized to the broader population of transition dairy cows. Further studies involving larger and more diverse populations are needed to confirm these observations and to further evaluate their potential application in precision dairy herd management.

## Figures and Tables

**Table 1:** Milk yield and milk composition across postpartum weeks (+1 to +5) (Mean ± SD).

Parameter	Week +1 (a)	Week +2 (b)	Week +3 (c)	Week +4 (d)	Week +5 (e)
Milk yield (kg/day)	17.9 (4.7) **c; *d,e	22.9 (8.93)	25.9 (9.83) **a	25.7 (6.29) *a	26.3 (6.87) **a
Fat (%)	5.62 (1.01) ***e;**d;*c	5.24 (1.04) **e;*d	4.74 (0.69) *a	4.41 (0.66) **a;*b	4.28 (0.72) ***a; **b
Protein (%)	3.98 (0.41) ***b,c,d,e	3.6 (0.31) ***a,c,d,e	3.37 (0.26) ***a,b;*d	3.19 (0.22) ***a,b;*c	3.15 (0.17) ***a,b
Lactose (%)	4.62 (0.21)	4.63 (0.24)	4.70 (0.33)	4.71 (0.23)	4.74 (0.17)
FPR	1.42 (0.18)	1.43 (0.21)	1.47 (0.31)	1.45 (0.25)	1.41 (0.24)

Letters indicate statistically significant pairwise differences between weeks according to Sidak-adjusted post hoc comparisons from the linear mixed model. Corresponding week letters are shown in parentheses in the column headings. Symbols indicate significance levels for the comparison with the referenced week (* *p* < 0.05; ** *p* < 0.01; *** *p* < 0.001). Absence of superscripts indicates no statistically significant difference.

**Table 2:** Blood biochemical parameters across weeks (−4 to +5) (Mean ± SD).

Parameter	Week −4 (a)	Week −3 (b)	Week −2 (c)	Week −1 (d)	Week +1 (e)	Week +2 (f)	Week +3 (g)	Week +4 (h)	Week +5 (i)
ALB (g/L)	35.5 (2.95)	34.8 (3.78)	34.7 (3.66)	35.2 (2.76)	32.6 (3.53)	33.3 (3.69)	31.8 (3.98)	34.1 (3.91)	32.7 (3.94)
ALT (U/L)	15.8 (3.2)	16.2 (3.5)	15.5 (3.1)	12.9 (2.8) *i	13.5 (3.0)	13.8 (3.2)	13.2 (2.9)	13.5 (3.1)	20.0 (4.5) *d
AST (U/L)	84.0 (12.4)	92.3 (44.1)	88.5 (38.0)	72.7 (11.8)	89.4 (24.3)	83.2 (14.5)	81.8 (18.9)	78.6 (17.9)	83.9 (15.1)
Ca (mmol/L)	2.48 (0.18)	2.50 (0.12)	2.61 (0.18) *g	2.51 (0.15)	2.47 (0.25)	2.40 (0.20)	2.37 (0.22) *c	2.48 (0.16)	2.37 (0.18)
CREA (µmol/L)	97.8 (25.3)	104.9 (30.1)	95.4 (33.2)	103 (23.9)	95.1 (26.7)	89.8 (23.0)	78.9 (17.5)	78.6 (21.1)	78.5 (19.3)
Fe (µmol/L)	22.3 (7.19) *c	23.9 (7.63) **c;*e,f	30.9 (10.1) ***e,f,g,h,i;*b,a	25.3 (8.9) ***e,f;**g	15.3 (8.5) ***c,d;*b	14.5 (6.01) ***c,d;*b	14.7 (5.62) ***c;**d;*b	16.8 (5.62) ***c	17.7 (5.49) ***c
GGT (U/L)	20.9 (5.32)	20.1 (4.31)	18.9 (7.13)	21.6 (5.04)	21.6 (5.86)	19.7 (6.48)	23.6 (6.54)	24.6 (5.07)	26.0 (7.22)
GLUC (mmol/L)	3.98 (0.62)	4.24 (0.39)	4.37 (0.43) **h	4.35 (0.74) **h	4.30 (0.46) *h	4.09 (0.45)	3.86 (0.52)	3.63 (0.43) **c,d;*e	3.78 (0.33)
Mg (mmol/L)	0.96 (0.15)	0.98 (0.18)	0.84 (0.11)	0.83 (0.11)	0.89 (0.14)	0.87 (0.09)	0.82 (0.16)	0.97 (0.10)	0.88 (0.25)
NEFA (mmol/L)	0.12 (0.05)	0.08 (0.04)	0.04 (0.02)	0.11 (0.05)	0.11 (0.05)	0.14 (0.06)	0.11 (0.05)	0.11 (0.05)	0.3 (0.12)
P (mmol/L)	2.19 (0.09) ***g;*f,i	2.34 (0.07) ***f	2.45 (0.15) ***f	2.24 (0.12) **f	2.95 (0.20) ***f,g;*i	1.41 (0.19) ***b,c,e;**d;*a,h	1.74 (0.12) ***a	2.04 (0.06) *f	2.0 (0.10) *a
TP (g/L)	72.3 (6.68)	68.5 (9.08)	68.1 (7.28)	68.0 (7.88)	67.9 (8.15)	73.8 (4.73)	74.3 (5.85)	75.8 (10.6)	79.3 (11.1)
TRIG (mmol/L)	0.2 (0.14) **d	0.2 (0.13) **d	0.19 (0.12) **d	0.11 (0.22) **a,b,c	0.13 (0.14)	0.14 (0.16)	0.12 (0.16)	0.13 (0.25)	0.18 (0.21)
UREA (mmol/L)	4.47 (0.10)	4.27 (0.09)	4.27 (0.10)	4.07 (0.19)	2.57 (0.21) ***f	7.08 (0.57) ***e	3.8 (0.10)	4.57 (0.07)	5.01 (0.06)

Letters indicate statistically significant pairwise differences between weeks according to Sidak-adjusted post hoc comparisons from the linear mixed model. Corresponding week letters are shown in parentheses in the column headings. Symbols indicate significance levels for the comparison with the referenced week (* *p* < 0.05; ** *p* < 0.01; *** *p* < 0.001). Absence of superscripts indicates no statistically significant difference.

**Table 3:** Changes in rumen physiology, behavioural parameters, water intake, and dry concentrate intake across weeks (−4 to +5) (Mean ± SD).

Parameter	Week −4 (a)	Week −3 (b)	Week −2 (c)	Week −1(d)	Week +1 (e)	Week +2 (f)	Week +3 (g)	Week +4 (h)	Week +5 (i)
Rumen temperature (°C)	39.1 (0.28)	39.2 (0.31)	39.1 (0.09)	39.5 (0.43) **h	39.1 (0.49)	39.1 (0.43)	39.2 (0.33)	38.9 (0.31) **d	39.2 (0.41)
Rumination time (min/day)	380 (92.4)	389 (81.49)	424 (60.9)	377 (85.9)	372 (81.3)	416 (109.9)	418 (71.8)	449 (80.1)	460 (90.7)
Activity (units 0–100)	10.8 (4.06) *f,g,h	10.6 (3.78) ***i,*f,g,h	11.1 (4.38) ***i;**f,g,h	11.5 (5.43) ***e,f,g,h,i	7.66 (3.44) ***d	6.08 (2.39) ***d;**a,b,c	5.75 (2.61) ***d;**f;*a,b	6.04 (3.09) ***d;**c;*a,b	4.66 (3.06) ***a,b,c,d
Water intake (L/day)	60.6 (27.1) **e,g	53.6 (15.4) ***e,g;**f,i;*h	61.8 (26.4) **e,g	82.1 (28.4)	99.4 (33.2) **a,b,c	93.1 (39.7) **b	104 (27.5) *c	92.9 (19.9) *b	96.0 (27.3) **b
Rumen pH	6.34 (0.25)	6.42 (0.27)	6.41 (0.37)	6.51 (0.28)	6.44 (0.41)	6.50 (0.42)	6.33 (0.41)	6.55 (0.35)	6.35 (0.39)
Concentrate intake (kg/day)	0.46 ***h, i; *g (0.08)	0.62 (0.11)	0.79 (0.12)	1.13 (0.33)	2.13 (0.64)	2.31 (0.33)	4.52 *a (1.06)	5.2 ***a (0.95)	5.74 ***a (1.02)

Letters indicate statistically significant pairwise differences between weeks according to Sidak-adjusted post hoc comparisons from the linear mixed model. Corresponding week letters are shown in parentheses in the column headings. Symbols indicate significance levels for the comparison with the referenced week (* *p* < 0.05; ** *p* < 0.01; *** *p* < 0.001). Absence of superscripts indicates no statistically significant difference.

**Table 4:** Linear mixed model summary for all dependent variables.

Variable	Weeks	F (WEEK)	df1	df2	*p* Value	AR(1) ρ	VAR (Random Intercept)	AR(1) Variance (Diagonal)
Milk yield (kg/day)	+1…+5	4.27	4	48.9	0.005	0.58	37.7	53.5
Fat (%)	+1…+5	5.52	4	45.8	0.001	0.53	0.5	0.7
Protein (%)	+1…+5	23.57	4	47.3	<0.001	0.73	0.00	0.08
Lactose (%)	+1…+5	0.79	4	50.1	0.533	0.66	0.00	0.05
FPR	+1…+5	1.1	4	46.5	0.36	0.46	7.4 × 10^−7^	0.01
ALB (g/L)	−4…+5	3.48	8	85.7	0.002	0.65	0.69	12.6
ALT (U/L)	−4…+5	3.04	8	48.7	0.007	0.08	0.00	0.07
AST (U/L)	−4…+5	1.16	8	77.6	0.333	0.31	0.00	587
Ca (mmol/L)	−4…+5	2.02	8	72.3	0.056	0.25	0.00	0.03
CREA (µmol/L)	−4…+5	2.07	8	71.4	0.050	0.18	36.5	616
Fe (µmol/L)	−4…+5	7.45	8	82.1	<0.001	0.53	0.00	53.4
GGT (U/L)	−4…+5	2.02	8	78.5	0.055	0.52	0.45	36.1
GLUC (mmol/L)	−4…+5	2.7	8	76.6	0.010	0.43	0.00	0.25
Mg (mmol/L)	−4…+5	2.42	8	62.7	0.024	0.12	0.00	0.02
NEFA (mmol/L)	−4…+5	0.94	8	67.2	0.483	0.19	0.00	0.30
P (mmol/L)	−4…+5	7.11	8	67.0	<0.001	–0.26	0.00	0.01
TP (g/L)	−4…+5	2.22	8	81.2	0.033	0.63	31.1	72.4
TRIG (mmol/L)	−4…+5	4.06	8	67.7	<0.001	0.11	0.00	0.03
UREA (mmol/L)	−4…+5	3.23	8	71.1	0.003	–0.09	0.01	0.05
Rumen temperature (°C)	−4…+5	2.31	8	69.5	0.029	0.18	0.02	0.13
Rumination time (min/24 h)	−4…+5	2.53	8	81.7	0.016	0.60	1346	7242
Rumen pH	−4…+5	2.18	8	84.1	0.037	0.61	0.00	0.12
Activity (units)	−4…+5	5.41	8	84.6	<0.001	0.63	0.5	13.4
Water intake (L/day)	−4…+5	4.22	8	78.1	<0.001	0.46	160	784
Concentrate intake (kg/day)	−4…+5	2.04	8	71.3	0.025	0.21	0.00	0.05

**Figure A1:**
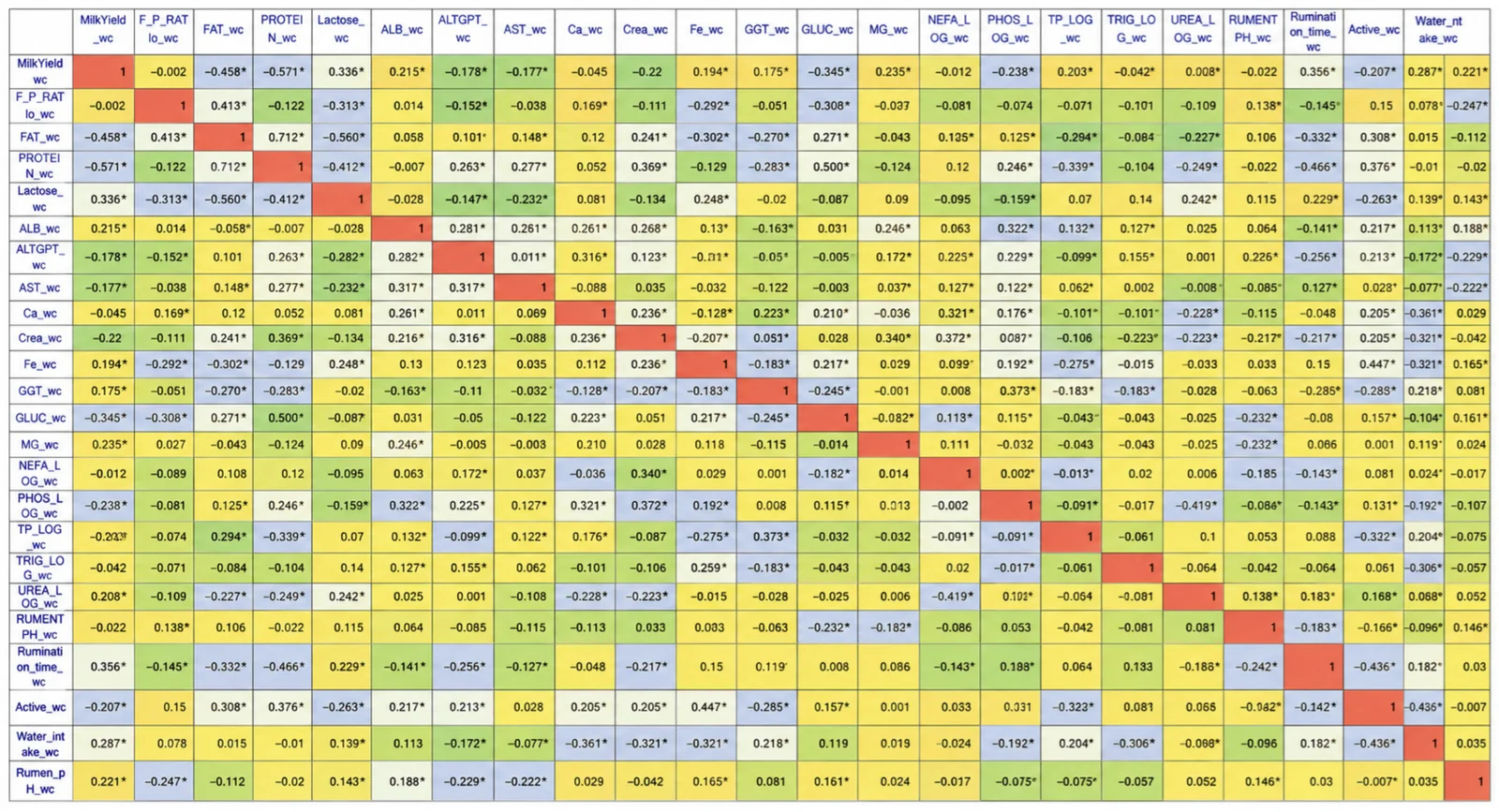
Correlation matrix of repeated-measures (within-cow) associations among milk production, behavioral, rumen, and serum biochemical variables in primiparous dairy cows during the transition period (weeks −4 to +5 relative to calving). Cell values represent repeated-measures correlation coefficients (rmcorr, r). Asterisks indicate statistically significant correlations (* *p* < 0.05).

## Data Availability

The data presented in this study are available on request from the corresponding author. The dataset comprises longitudinal measurements at the individual cow level, including weekly production data, sensor-derived behavioural variables, and blood biochemical parameters used for statistical analyses. Due to data ownership agreements and farm-level confidentiality restrictions, the data cannot be made publicly available. However, all relevant data required to replicate the analyses and support the findings of this study can be provided upon request.
